# Supplementary material for: GiRAFR improves gRNA detection and annotation in single-cell CRISPR screens
Source: Commun Biol. 2023 Sep 23;6:975. doi: 10.1038/s42003-023-05351-7 (PMC10518011; doi:10.1038/s42003-023-05351-7)
Supplement: Supplementary file 2 — Supplementary Information [file 42003_2023_5351_MOESM2_ESM.pdf]

Supplementary Note 1:

CIGAR-like string:

Digit numbers represents exact matches, and nucleotides followed are mutated bases. 0 represents no nucleotide.

Digit numbers followed by insertions (I), deletions (D) and soft clippings (S) show the number of nucleotides of those events. Hard clippings (H) are not included. The major difference between this string and CIGAR-string is it replaces matches (M) into mismatches and encode detailed mutated nucleotides [ATGC] into the string.

Mutation structure annotation:

Annotations begin with oligo structures such as gRNA which are consistent with user input oligo pool plasmid structure.gtf. Then each mutation annotation follows oligo structure with semicolon as separator. Comma separates individual mutation event. Digit numbers represents the distance to the beginning of the structure. Nucleotides followed are mutated bases. 0 represents no nucleotide. Digit numbers in bracket followed insertions (I), deletions (D) and soft clippings (S) represent the number of nucleotides of those events.

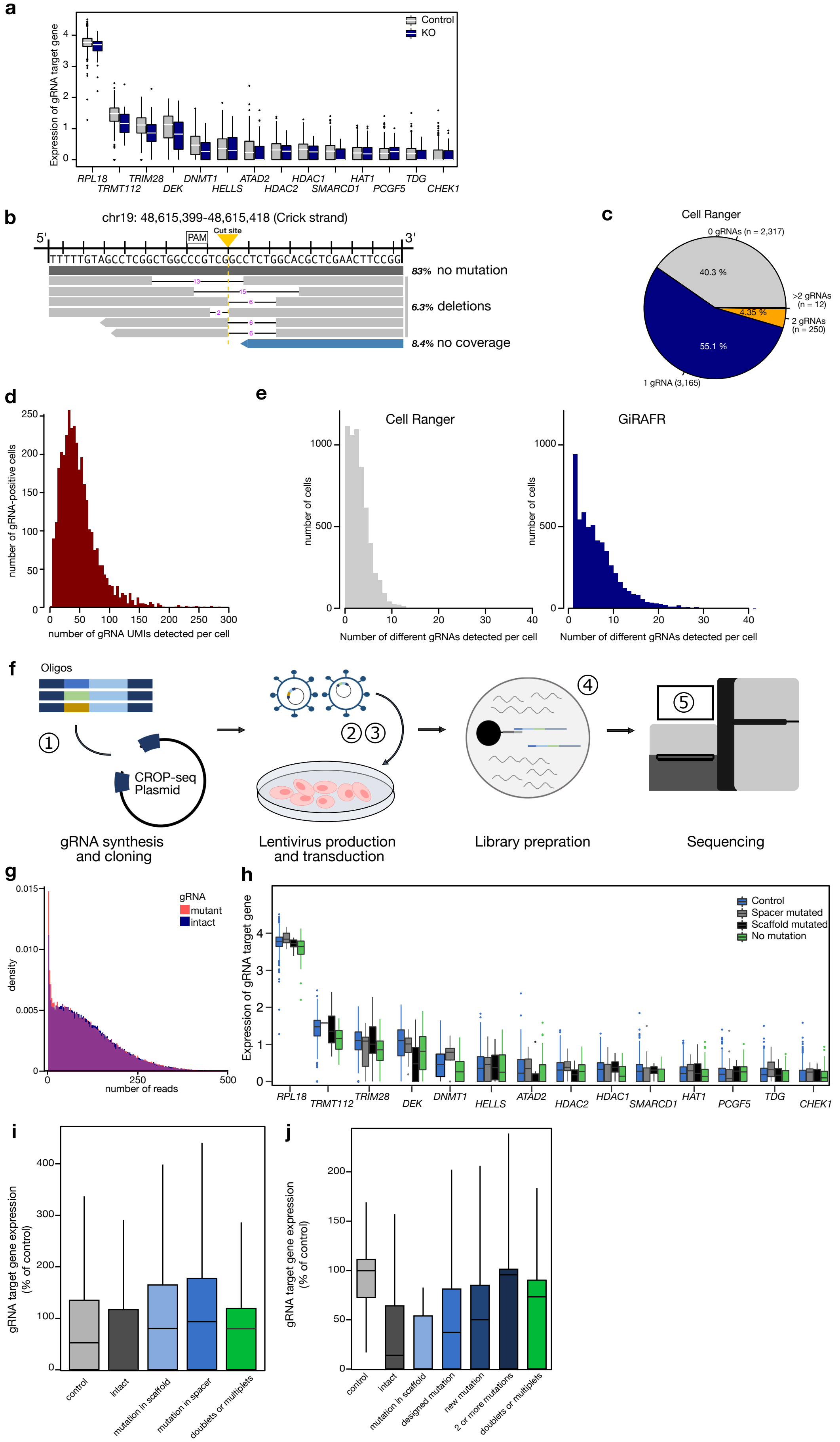
a
Control
KO
Expression of gRNA target gene
RPL18 TRMT112 TRIM28 DEK DNMT1 HELLS ATAD2 HDAC2 HDAC1 SMARCD1 HAT1 PCGF5 TDG CHEK1
b
chr19: 48,615,399-48,615,418 (Crick strand)
5'
PAM
Cut site
3'
TTTTTGTAGCCTCGGCTGGCCCGTCGGCCTCTGGCACGCTCGAACTTCCGG
83% no mutation
6.3% deletions
8.4% no coverage
c
Cell Ranger
0 gRNAs (n = 2,317)
40.3 %
4.35 %
55.1 %
>2 gRNAs (n = 12)
2 gRNAs (n = 250)
1 gRNA (3,165)
d
number of gRNA-positive cells
number of gRNA UMIs detected per cell
e
Cell Ranger
GiRAFR
number of cells
Number of different gRNAs detected per cell
f
Oligos
CROP-seq Plasmid
gRNA synthesis and cloning
Lentivirus production and transduction
Library prepration
Sequencing
g
gRNA
mutant
intact
density
number of reads
h
Control
Spacer mutated
Scaffold mutated
No mutation
Expression of gRNA target gene
RPL18 TRMT112 TRIM28 DEK DNMT1 HELLS ATAD2 HDAC2 HDAC1 SMARCD1 HAT1 PCGF5 TDG CHEK1
i
gRNA target gene expression (% of control)
control
intact
mutation in scaffold
mutation in spacer
doublets or multiplets
j
gRNA target gene expression (% of control)
control
intact
mutation in scaffold
designed mutation
new mutation
2 or more mutations
doublets or multiplets

Supplementary Figure 1: GiRAFR detects mutant gRNAs from in-house CROP-seq experiment. a. Expression (log-transformed read counts) of the 14 gRNA target genes showing expression in at least 50 % of all control cells. Boxplot denotes quartile range (box), median (center line) and 1.5x interquartile range (whiskers). **b.** 51 bp window of RPL18, showing the predicted cut site of gRNA 1 (targets the Crick strand). Nine cells expressing this gRNA show reads coverage in this detection window. 6 of 95 reads (6.3 %) with a unique UMI show a deletion across the cut site (3-4bp upstream of PAM), 83% show no mutations, and 8.4% have no coverage over the cut site. Note that transcripts with out-of-frame indels are likely subject to nonsense-mediated decay. These frequencies therefore likely underestimate the genome editing efficiency. **c.** Pie chart showing the number of cells with 0, 1, 2 or more than 2 gRNAs assigned to them by Cell Ranger. **d.** Histogram showing the number of different gRNA UMIs detected in gRNA-positive cells by Cell Ranger. **e.** Histograms showing the number of different gRNAs detected by Cell Ranger and GiRAFR. Note that GiRAFR discards all cells in which no gRNA is detected. For Cell Ranger, only gRNAs matching the designed sequence are considered, while for GiRAFR, both intact and mutant gRNAs may be counted. **f.** Schematic overview of CROP-seq experiment with different sources of gRNA mutations. ①: Inaccurate oligonucleotide synthesis ②: Errors during lentivirus transduction ③: gRNA cassette genome integration ④: Errors induced during gRNA transcription or during cDNA synthesis ⑤: Sequencing errors **g.** Density histogram showing read number of UMIs in mutant and intact gRNA **h.** Expression (log-transformed read counts) of 14 gRNA target genes which show expression in at least 50 % of all control cells. Boxplot denotes quartile range (box), median (center line) and 1.5x interquartile range (whiskers). 'intact' bar shows the expression in cells with intact gRNAs. 'mutation in scaffold', 'mutation in spacer' show the novel mutations identified by GiRAFR with mutations in the spacer region and gRNA spacer region. 'doublets or multiplets' show the cells with more than 1 gRNA newly identified by GiRAFR. **i.** Box plot of average expression

(log-transformed read counts) of gRNA target genes, normalized to their expression in cells with non-targeting gRNAs (control cells) using in-house CROP-seq data. Box plot denotes quartile range (box), median (center line) and 1.5x interquartile range (whiskers). **j.** Similar as **i** using data from Jost *et al.* ‘designed mutation’ bar shows the expression of single mismatch gRNAs as designed in the experiment. ‘mutation in scaffold’, ‘new mutation’ and ‘2 or more mutations’ show the novel mutations identified by GiRAFR with 0, 1 and 2 or more mutations in the spacer region.

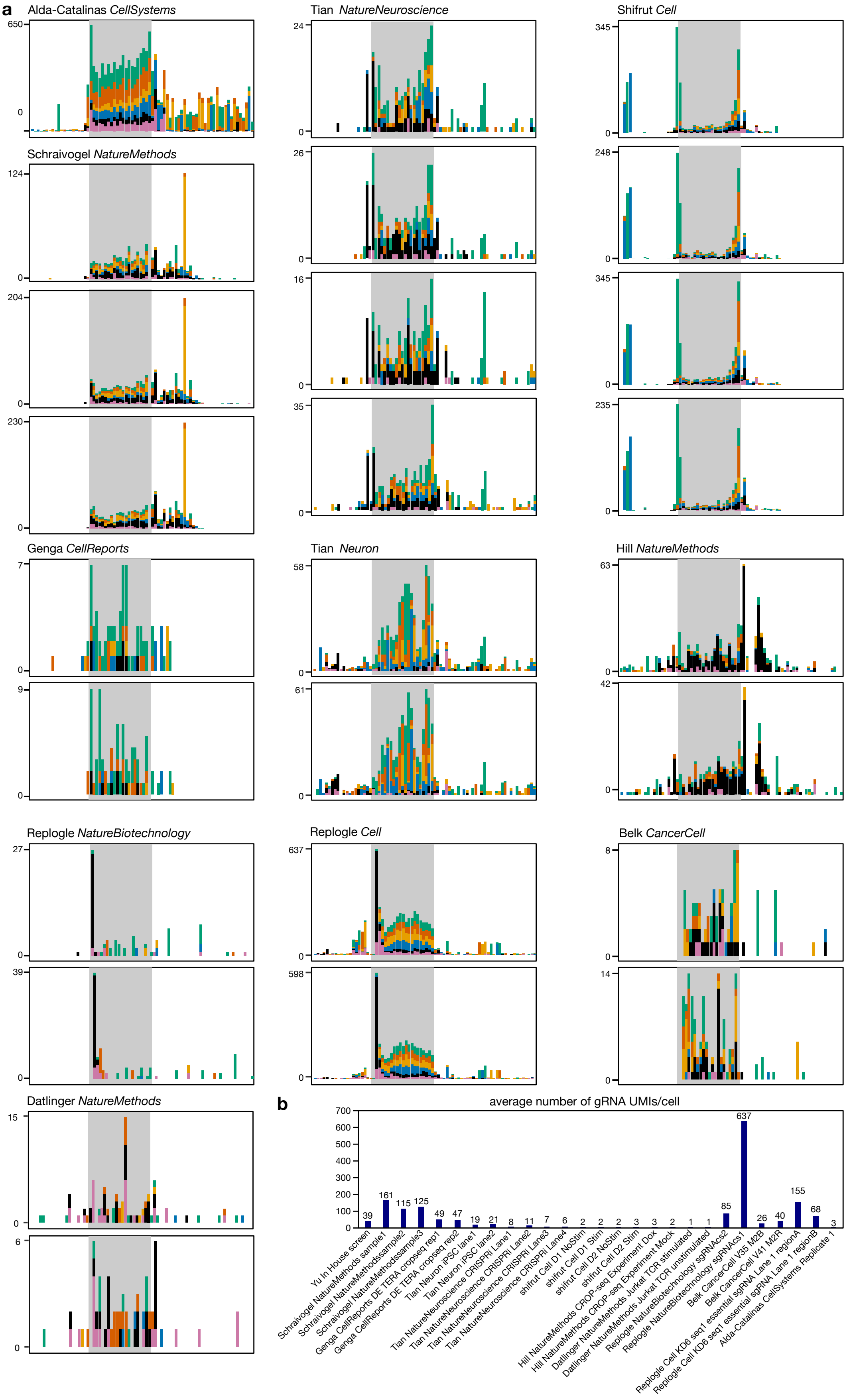
a
Alda-Catalinas CellSystems
650
0
Tian NatureNeuroscience
24
0
Shifrut Cell
345
0
Schraivogel NatureMethods
124
0
26
0
248
0
204
0
16
0
345
0
230
0
35
0
235
0
Genga CellReports
7
0
Tian Neuron
58
0
Hill NatureMethods
63
0
9
0
61
0
42
0
Replogle NatureBiotechnology
27
0
Replogle Cell
637
0
Belk CancerCell
8
0
39
0
598
0
14
0
Datlinger NatureMethods
15
0
6
0
b
average number of gRNA UMIs/cell
700
600
500
400
300
200
100
0
39
161
115
125
49
47
19
21
8
11
7
6
2
2
2
3
3
2
1
1
85
637
26
40
155
68
3
Yu In House screen
Schraivogel NatureMethods sample1
Schraivogel NatureMethodssample2
Schraivogel NatureMethodssample3
Genga CellReports DE TERA cropseq rep1
Genga CellReports DE TERA cropseq rep2
Tian Neuron iPSC lane1
Tian Neuron iPSC lane2
Tian NatureNeuroscience CRISPRi Lane1
Tian NatureNeuroscience CRISPRi Lane2
Tian NatureNeuroscience CRISPRi Lane3
Tian NatureNeuroscience CRISPRi Lane4
shifrut Cell D1 NoStim
shifrut Cell D1 Stim
shifrut Cell D2 NoStim
shifrut Cell D2 Stim
Hill NatureMethods CROP-seq Experiment Dox
Hill NatureMethods CROP-seq Experiment Mock
Datlinger NatureMethods Jurkat TCR stimulated
Datlinger NatureMethods Jurkat TCR unstimulated
Replogle NatureBiotechnology sgRNAcs2
Replogle NatureBiotechnology sgRNAcs1
Belk CancerCell V35 M2B
Belk CancerCell V41 M2R
Replogle Cell KD6 seq1 essential sgRNA Lane 1 regionA
Replogle Cell KD6 seq1 essential sgRNA Lane 1 regionB
Alda-Catalinas CellSystems Replicate 1

Supplementary Figure 2: Mutation spectra and sequencing depth of analyzed samples. **a.** Mutation frequency of gRNAs associated with cells after the Gaussian Mixture model filtering. Shown are all gRNA mutation patterns detected in publicly available datasets. Note that some gRNA libraries encompass only a fraction of the U6 promoter shown. The aggregate of these experiments per study is shown in Figure 3a. Striped boxes indicate the position of template switching oligonucleotides, where mutations on position -2, -1 and 0 before the start of the spacer were also removed. Such mutations are likely produced by the terminal nucleotidyl transferase activity of the reverse transcriptase, and hence not present in the gRNA. **b.** The average number of gRNA UMIs per cell, as determined by GiRAFR.

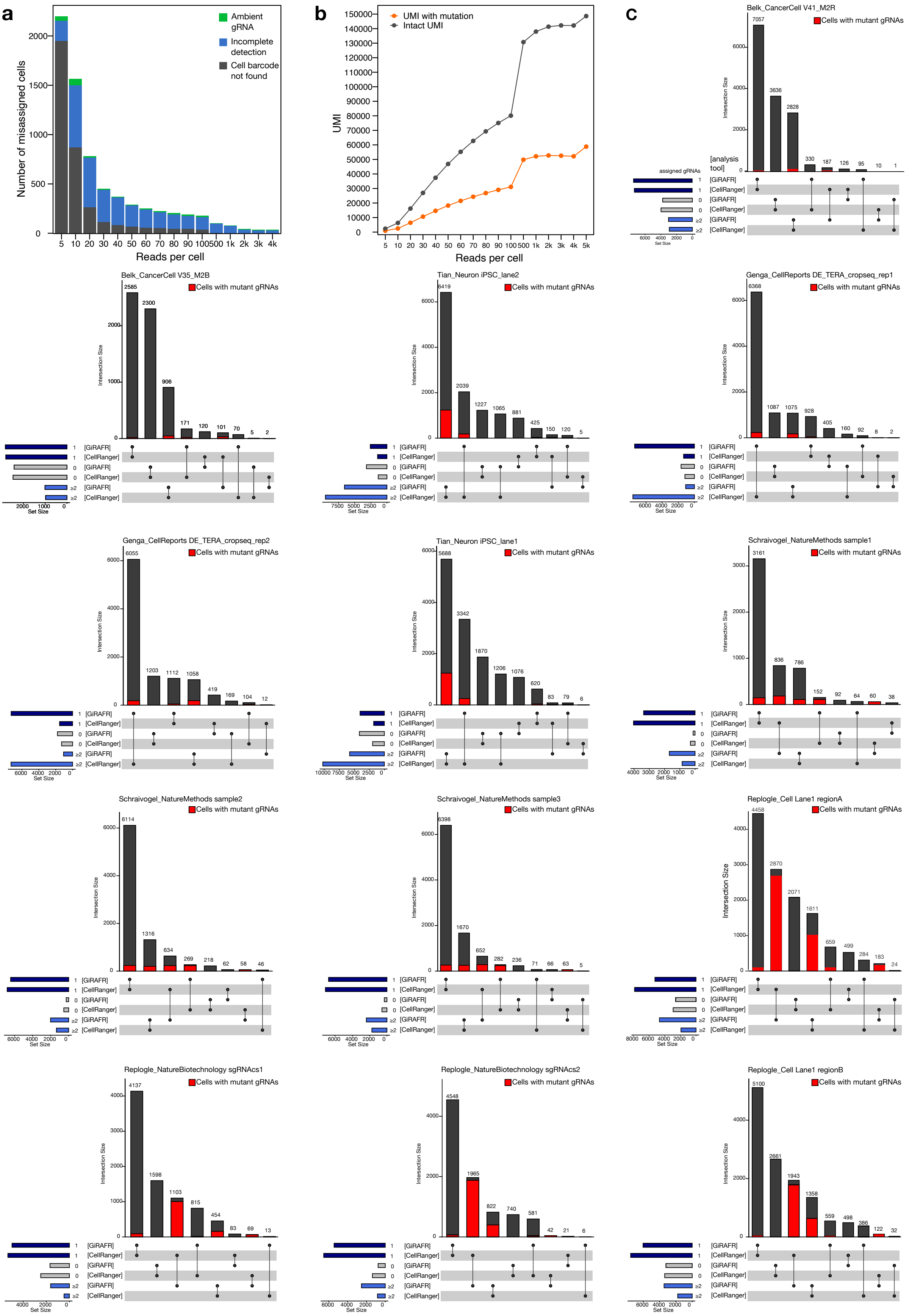

a
Ambient gRNA
Incomplete detection
Cell barcode not found
Number of misassigned cells
Reads per cell
b
UMI with mutation
Intact UMI
UMI
Reads per cell
c
Belk_CancerCell V41_M2R
Cells with mutant gRNAs
Intersection Size
assigned gRNAs
[analysis tool]
[GiRAFR]
[CellRanger]
Set Size
Belk_CancerCell V35_M2B
Tian_Neuron iPSC_lane2
Genga_CellReports DE_TERA_cropseq_rep1
Genga_CellReports DE_TERA_cropseq_rep2
Tian_Neuron iPSC_lane1
Schraivogel_NatureMethods sample1
Schraivogel_NatureMethods sample2
Schraivogel_NatureMethods sample3
Replogle_Cell Lane1 regionA
Replogle_NatureBiotechnology sgRNAcs1
Replogle_NatureBiotechnology sgRNAcs2
Replogle_Cell Lane1 regionB

Supplementary Figure 3: Sequencing saturation impacts mutation calling and cell assignments of GiRAFR compared with Cell Ranger of high-depth experiments. a. Number of inaccurate assignments in down-sampled inhouse CROP-seq experiment. Compared to full data (5k reads per cell), mis-assignments are categorized as no gRNA could be detected (grey), not all expressed gRNAs were detected (blue), and wrong gRNA assigned (green). **b.** Number of detected UMIs in down-sampled inhouse CROP-seq experiment gRNA library. **c.** Comparison of gRNA assignment to cells between Cell Ranger and GiRAFR (25). The red box indicates cells containing 1 or more mutant gRNAs. Shown are data for each of the 13 experiments for which on average over 16 unique gRNA molecules were recovered per cell, suggesting that over 95 % of cells are correctly assigned.

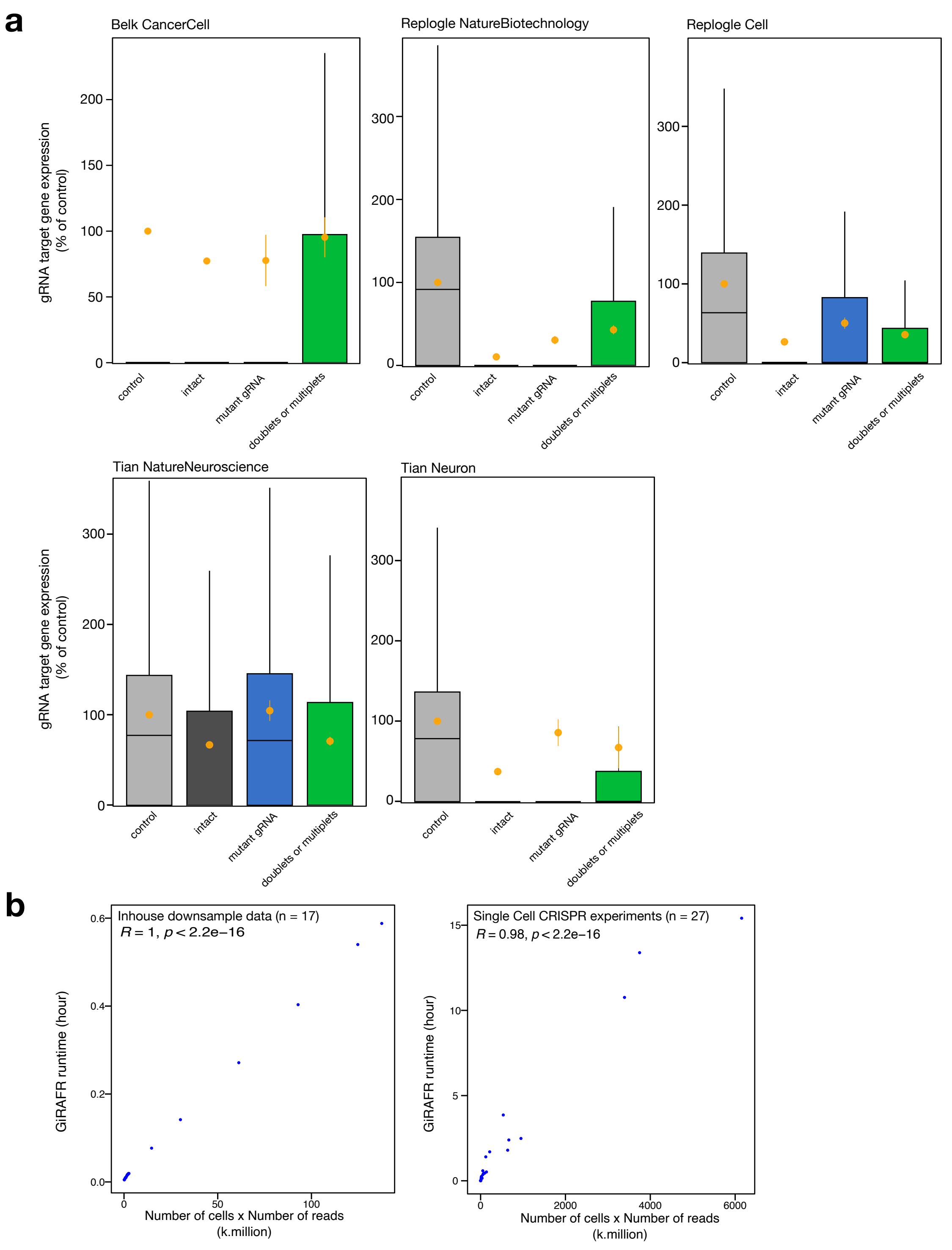

Supplementary Figure 4: Attenuated functionality of mutant gRNAs and scalability of GiRAFR. a. Box plots of average expression (log-transformed read counts) of gRNA target genes, normalized to their expression in cells with non-targeting gRNAs (control cells) using data from selected high sequencing depth experiments. Box plot denotes quartile range (box), median (center line) and 1.5x interquartile range (whiskers). Yellow dot and line indicate

average value ± standard error of mean. ‘mutant gRNA’ bar shows the cells with mutant gRNA. ‘doublets or multiplets’ show the cells which were identified by GiRAFR as doublets or multiple’s but singlets by Cell Ranger. **b**. Correlation between GiRAFR runtime and the product of cells number and reads number using inhouse down sample data (left) and publicly available single cell CRISPR experiments (right). Shown are Pearson correlations and runtime of input bam file cleaning and preparation was not included.

gRNA name	gRNA spacer sequence	target gene EnsID	target gene HGNC symbol
ATAD2_gRNA1	CGCGGCGCAGAAGAAACCCG	ENSG00000156802	*ATAD2*
ATAD2_gRNA2	AGGAAGTTGAAACCTACCAC	ENSG00000156802	*ATAD2*
ATAD2_gRNA3	TAGGCAGTTGGCCAGACAGC	ENSG00000156802	*ATAD2*
ATAD2_gRNA4	ATGAAGTCGAGTCACTACTG	ENSG00000156802	*ATAD2*
CHEK1_gRNA1	TTGGTGCAAACCCTGGGAGA	ENSG00000149554	*CHEK1*
CHEK1_gRNA2	CTTCCATCAACTCATGGCAG	ENSG00000149554	*CHEK1*
CHEK1_gRNA3	GGAATGGTAATTCTTGCTGA	ENSG00000149554	*CHEK1*
CHEK1_gRNA4	GGTGTGTCAGAGTCTCCCAG	ENSG00000149554	*CHEK1*
DEK_gRNA1	TCCCAGAGAGGAGAGCGAGG	ENSG00000124795	*DEK*
DEK_gRNA2	GAGCCATTTACAATTGCACA	ENSG00000124795	*DEK*
DEK_gRNA3	AAACTGCTTTACAACAGGCC	ENSG00000124795	*DEK*
DEK_gRNA4	AAGAATGTGGGTCAGTTCAG	ENSG00000124795	*DEK*
DHODH_gRNA1	GACCTCAGTGGACGCCGCGG	ENSG00000102967	*DHODH*
DHODH_gRNA2	TGACAGCTTGGTCCTCAGGG	ENSG00000102967	*DHODH*
DHODH_gRNA3	ATGACGGAAGGAGCATGGCG	ENSG00000102967	*DHODH*
DHODH_gRNA4	GAGTCTTGAAATCTGGCCCG	ENSG00000102967	*DHODH*
DNMT1_gRNA1	GGGCAGCGAGATGGCCGGGA	ENSG00000130816	*DNMT1*
DNMT1_gRNA2	AAAGGGATTTGACTTTAGCC	ENSG00000130816	*DNMT1*
DNMT1_gRNA3	GGAGCCGGACAGAGAAGCCA	ENSG00000130816	*DNMT1*
DNMT1_gRNA4	CCTGCAGAGCTGCCAAACGG	ENSG00000130816	*DNMT1*
DNMT3A_gRNA1	GCTACCACGCCTGAGCCCGT	ENSG00000119772	*DNMT3A*
DNMT3A_gRNA2	GAGCAGCTGAAGGCACCCGC	ENSG00000119772	*DNMT3A*
DNMT3A_gRNA3	GTACCGCAAAGCCATCTACG	ENSG00000119772	*DNMT3A*
DNMT3A_gRNA4	GAGTGACACTGCCAAGGCCG	ENSG00000119772	*DNMT3A*
DNMT3B_gRNA1	AATGGAGAGGAGGACGCCGG	ENSG00000088305	*DNMT3B*
DNMT3B_gRNA2	AAGCTCGCGACTCTCCAAGA	ENSG00000088305	*DNMT3B*
DNMT3B_gRNA3	GACTTGACAGGCGATGGCGA	ENSG00000088305	*DNMT3B*
DNMT3B_gRNA4	GGTAGCCGGGAACTCCACGG	ENSG00000088305	*DNMT3B*
EZH2_gRNA1	ACTGGGAAGAAATCTGAGAA	ENSG00000106462	*EZH2*
EZH2_gRNA2	ACCAAGAATGGAAACAGCGA	ENSG00000106462	*EZH2*
EZH2_gRNA3	GATCTGGAGGATCACCGAGA	ENSG00000106462	*EZH2*
EZH2_gRNA4	TCAGAAGGAAATTTCCGAGG	ENSG00000106462	*EZH2*
HAT1_gRNA1	GGTAGAATATAAGAGTGCAG	ENSG00000128708	*HAT1*
HAT1_gRNA2	TCAGTTCTCAGTCCAACAGG	ENSG00000128708	*HAT1*
HAT1_gRNA3	ACTGCCAATTACCTTACACG	ENSG00000128708	*HAT1*
HAT1_gRNA4	TTTCAAGAAGTTGAGCACCA	ENSG00000128708	*HAT1*
HDAC1_gRNA1	GAGCAAGATGGCGCAGACGC	ENSG00000116478	*HDAC1*
HDAC1_gRNA2	CCCTCACAAAGCCAATGCTG	ENSG00000116478	*HDAC1*
HDAC1_gRNA3	GCACCATGCAAAGAAGTCCG	ENSG00000116478	*HDAC1*
HDAC1_gRNA4	TAAAGGGCGTTCTCACCCGT	ENSG00000116478	*HDAC1*
HDAC2_gRNA1	ATGGCGTACAGTCAAGGAGG	ENSG00000196591	*HDAC2*
HDAC2_gRNA2	AAACCGACAACAGACTGATA	ENSG00000196591	*HDAC2*
HDAC2_gRNA3	GATGTATCAACCTAGTGCTG	ENSG00000196591	*HDAC2*
HDAC2_gRNA4	GATAAATCCAAGGACAACAG	ENSG00000196591	*HDAC2*
HELLS_gRNA1	TGAGTGTCCAGGCATGCCAG	ENSG00000119969	*HELLS*
HELLS_gRNA2	AGGCTCGGAGGCTCCAGCAA	ENSG00000119969	*HELLS*
HELLS_gRNA3	GATGCAAGTGAAGAGAAGCC	ENSG00000119969	*HELLS*
HELLS_gRNA4	TCACTGGAGGAGTGATGCGA	ENSG00000119969	*HELLS*
KAT2B_gRNA1	GCGAGCGCGTACCTTGCAGG	ENSG00000114166	*KAT2B*
KAT2B_gRNA2	TTAGGAACTCACCTAGGGCA	ENSG00000114166	*KAT2B*
KAT2B_gRNA3	TATGAAATTGTCCCAGCCAC	ENSG00000114166	*KAT2B*
KAT2B_gRNA4	TCCGATGGAATTAATCAACG	ENSG00000114166	*KAT2B*
MTF2_gRNA1	TCTAAACAGAGACTCTACAG	ENSG00000143033	*MTF2*
MTF2_gRNA2	AATGTCCTTCCAGAGAACCC	ENSG00000143033	*MTF2*
MTF2_gRNA3	GCAGACATTACCCTATAGTG	ENSG00000143033	*MTF2*
MTF2_gRNA4	AGTGCTGCAAATGTAAGCAG	ENSG00000143033	*MTF2*
MVD_gRNA1	AAGCGGAGGAACTCACGGGA	ENSG00000167508	*MVD*
MVD_gRNA2	CATCAGCAAGGACTTCACCG	ENSG00000167508	*MVD*
MVD_gRNA3	GCGCGGTGGGACCATGGCCT	ENSG00000167508	*MVD*
MVD_gRNA4	GTGCAGAGTGACGCTCAGGG	ENSG00000167508	*MVD*
Non-Targeting_gRNA1	GGTGATCCTAGTCGACTGGC	Non-Targeting	Non-Targeting
Non-Targeting_gRNA2	GCTATTGTCGGCTGGATGGA	Non-Targeting	Non-Targeting
Non-Targeting_gRNA3	GCGCATCAATATGCCCGCAC	Non-Targeting	Non-Targeting
Non-Targeting_gRNA4	GAGGTCCCGCCTCCGCCCAA	Non-Targeting	Non-Targeting
Non-Targeting_gRNA1	GGCTAACGCACGCCGAGGTG	Non-Targeting	Non-Targeting

Non-Targeting_gRNA2	GGGCCTTAATAGTTCAACGC	Non-Targeting	Non-Targeting
Non-Targeting_gRNA3	GCAGGACGATAGTAACGTCC	Non-Targeting	Non-Targeting
Non-Targeting_gRNA4	GAGAACGTGATAAGACTCGG	Non-Targeting	Non-Targeting
Non-Targeting_gRNA1	GGACTGACGTATACGCTTGC	Non-Targeting	Non-Targeting
Non-Targeting_gRNA2	GTGAATACCCGTGACGGACA	Non-Targeting	Non-Targeting
Non-Targeting_gRNA3	GGCAATCGGCACGGCAAGGT	Non-Targeting	Non-Targeting
Non-Targeting_gRNA4	GGGCTGTTCTCACTCGTAGC	Non-Targeting	Non-Targeting
Non-Targeting_gRNA1	GGTTAAAGAGATTGTACGCC	Non-Targeting	Non-Targeting
Non-Targeting_gRNA2	GGGTTGCACGTAGGATATTC	Non-Targeting	Non-Targeting
Non-Targeting_gRNA3	GCGTATTGTATCCGCCACCG	Non-Targeting	Non-Targeting
Non-Targeting_gRNA4	GAGTAAAGCCGATTATATCG	Non-Targeting	Non-Targeting
Non-Targeting_gRNA1	GACCCTGAACAACCTAGGAT	Non-Targeting	Non-Targeting
Non-Targeting_gRNA2	GGACCGGCACCTTATTATAT	Non-Targeting	Non-Targeting
Non-Targeting_gRNA3	GAATGCTGAGTACGGTCTGT	Non-Targeting	Non-Targeting
Non-Targeting_gRNA4	GGTCTATGGCCACCCGCTAC	Non-Targeting	Non-Targeting
PCGF5_gRNA1	AGCCACGAATGGCTACCCAA	ENSG00000180628	*PCGF5*
PCGF5_gRNA2	GATCAAGCCAACAACAGTGA	ENSG00000180628	*PCGF5*
PCGF5_gRNA3	AAGATAGCAATGATTGCCCA	ENSG00000180628	*PCGF5*
PCGF5_gRNA4	AAGTTGGATGTGCTGTGCAA	ENSG00000180628	*PCGF5*
RPL18_gRNA1	TCGAGCGTGCCAGAGGCCGA	ENSG00000063177	*RPL18*
RPL18_gRNA2	CCTTCCCTAGGTCCTCGCAA	ENSG00000063177	*RPL18*
RPL18_gRNA3	AGTGGACATCCGCCATAACA	ENSG00000063177	*RPL18*
RPL18_gRNA4	CAGCCGCATCCTCAGGGCAG	ENSG00000063177	*RPL18*
SMARCD1_gRNA1	CAGTCTGTGGCTCCAAGCGG	ENSG00000066117	*SMARCD1*
SMARCD1_gRNA2	AGGCAGCCGAATGACACCTC	ENSG00000066117	*SMARCD1*
SMARCD1_gRNA3	GAAACGGCTAGATATCCAAG	ENSG00000066117	*SMARCD1*
SMARCD1_gRNA4	GGTAGAAGGACGGCTCCTGG	ENSG00000066117	*SMARCD1*
SUV39H1_gRNA1	GTTCCTCTTAGAGATACCGA	ENSG00000101945	*SUV39H1*
SUV39H1_gRNA2	TAGAAAGGGAGCTGCTCCGG	ENSG00000101945	*SUV39H1*
SUV39H1_gRNA3	CGTGGAGGACGTGTACACCG	ENSG00000101945	*SUV39H1*
SUV39H1_gRNA4	TCATAGACAACCTTGACGAG	ENSG00000101945	*SUV39H1*
TDG_gRNA1	GGAATGGAAGCGGAGAACGC	ENSG00000139372	*TDG*
TDG_gRNA2	TACAAAGGGCATCATTACCC	ENSG00000139372	*TDG*
TDG_gRNA3	TGGATGATCACACTCTACCA	ENSG00000139372	*TDG*
TDG_gRNA4	TTTGACCTACAGCTTGCCCA	ENSG00000139372	*TDG*
TET1_gRNA1	GAGACATTGAATGATACCAG	ENSG00000138336	*TET1*
TET1_gRNA2	ACAAAGTTCATGCAACACGG	ENSG00000138336	*TET1*
TET1_gRNA3	GGAAGGTCGTCCCTTCTCTG	ENSG00000138336	*TET1*
TET1_gRNA4	GGAAGCCAAGATCAAATCTG	ENSG00000138336	*TET1*
TRIM28_gRNA1	GTGAATGGCGGCCTCCGCGG	ENSG00000130726	*TRIM28*
TRIM28_gRNA2	GAAGCACTGTTGCTTGCACA	ENSG00000130726	*TRIM28*
TRIM28_gRNA3	CCAGCGGGTGAAGTACACCA	ENSG00000130726	*TRIM28*
TRIM28_gRNA4	ATTGAGCTGGCAGTCTCGGC	ENSG00000130726	*TRIM28*
TRMT112_gRNA1	GATCCAGGTGCCGAAAGGGC	ENSG00000173113	*TRMT112*
TRMT112_gRNA2	AGGGCAGATACGGACCTCGG	ENSG00000173113	*TRMT112*
TRMT112_gRNA3	GCTGAGCTCGCATGTGCGGG	ENSG00000173113	*TRMT112*
TRMT112_gRNA4	TGCTGAGCTCGCATGTGCGG	ENSG00000173113	*TRMT112*
TUBB1_gRNA1	GGAAGATGAAGAGGTCACGG	ENSG00000101162	*TUBB1*
TUBB1_gRNA2	GCAGTACCGAGCCCTCTCCG	ENSG00000101162	*TUBB1*
TUBB1_gRNA3	ACTTGGCTGGGAGCGACCGC	ENSG00000101162	*TUBB1*
TUBB1_gRNA4	TGTGGTGGAGCCCTACAACG	ENSG00000101162	*TUBB1*
UHRF1_gRNA1	TGCTCGGGACACGAACATGG	ENSG00000276043	*UHRF1*
UHRF1_gRNA2	GATCCAGGAGCTGTTCCACG	ENSG00000276043	*UHRF1*
UHRF1_gRNA3	ACACCAGTGGACACAGATGG	ENSG00000276043	*UHRF1*
UHRF1_gRNA4	TGGCCTGCTGTCAGTCTCGG	ENSG00000276043	*UHRF1*

Supplementary Table 1: 120 gRNAs used in the CROP-seq experiments in this study.

Shown columns are unique name for each gRNA used in data analysis, designed gRNA spacer sequence, target gene Ensembl ID and HGNC symbol.

	REF	Dataset	CROP-seq vector	Constant sequence used for feature barcoding	gRNA length	scRNAseq method	gRNA read (bp)	gRNA capture method
this study		GSE216040	CROPseq-Guide-Puro Addgene #86708	TGGAAAGGAC GAAACACCG (BC)	G + 20bp	Single Cell 3' v3.1 kit	91	polyA tail
Schraivogel Nature Methods	(10)	GSM4012694; GSM4012695; GSM4012696	CROPseq-Guide-Puro Addgene #86708 and CROPseq-Puro-F+E	TGGAAAGGAC GAAACACCG (BC)	G + 19bp most gRNAs	Single Cell 3' v2 kit	58	polyA tail
Genga Cell Reports	(8)	GSM3630200; GSM3630201; GSM3630202; GSM3630203	CROPseq-Guide-Puro Addgene #86708	TGGAAAGGAC GAAACACC (BC)	G + 19bp	Single Cell 3' v2 kit	50	polyA tail
Tian Neuron	(11)	GSM3543620; GSM3543621	Adapted CROP-Seq pMK1334 vector Addgene #127965	TTGGAGAAC CACCTTGTTG (BC)	G + 20bp	Single Cell 3' v2 kit	90	Capture sequence in gRNA 3'-end
Tian Nature Neuroscience	(7)	GSM4632022; GSM4632023; GSM4632025; GSM4632026	Adapted CROP-Seq pMK1334 vector Addgene #127965	/	G + 20bp	Single Cell 3' v3.1 kit	91	Capture sequence in gRNA 3'-end
Shifrut Cell	(6)	GSM3375487; GSM3375488; GSM3375489; GSM3375490	CROPseq-Guide-Puro Addgene #86708	/	G + 20bp	Single Cell 3' v2 kit	58	polyA tail
Hill Nature Methods	(12)	GSM2911346; GSM2911347	CROPseq-Guide-Puro Addgene #86708	/	G + 20bp	Single Cell 3' v1 kit	64	polyA tail
Datlinger Nature Methods	(4)	GSM2439080: GSM2439085; GSM2439086; GSM2439090	CROPseq-Guide-Puro Addgene #86708	/	G + 20bp	Drop-Seq	64	polyA tail
Replogle Nature Biotechnology	(3)	GSM4367980; GSM4367981	pBA904, Addgene #122238 and pBA900, Addgene #122237	CAACGCAGAG TACATGGG (BC)	G + 18/19/20bp	Single Cell 3' v3.1 kit	125	Capture sequence at sgRNA 3'-end or in stem loop 2
Belk Cancer Cell	(13)	GSM6176619; GSM6176629	Addgene #104861	TTTCTTATAT GGGG (BC)	G + 20bp	Single Cell V(D)J Reagent Kits 5' v1.1	91	KP bead sgRNA RT primer during GEM generation
Replogle Cell	(14)	SRR19330404; SRR19330632; SRR19330643; SRR19330707	pRS275/pJR101 vector	CAACGCAGAG TACATGGG (BC)	G + 20bp	Single Cell 3’ v3 kit	98	Feature barcoding: Capture sequence
Alda-Catalinas Cell Systems	(9)	GSM4023607	CROP-sgRNA-MS2 Addgene plasmid #153457	/	G + 20bp	Single Cell 3' v2 kit	267	Feature barcoding: Capture sequence

Supplementary Table 2: Summary table of datasets used in this study. This table includes 1 new CROP-seq data and 26 single cell CRISPR experiments from 11 studies. Constant sequence is only given when Cell Ranger feature barcoding analysis can be applied.
